# Effect of Modest Caloric Restriction on Oxidative Stress in Women, a Randomized Trial

**DOI:** 10.1371/journal.pone.0047079

**Published:** 2012-10-05

**Authors:** Maciej S. Buchowski, Nobuko Hongu, Sari Acra, Li Wang, Joshua Warolin, L. Jackson Roberts

**Affiliations:** 1 Department of Medicine, Division of Gastroenterology, Hepatology and Nutrition, Vanderbilt University School of Medicine, Nashville, Tennessee, United States of America; 2 Department of Nutritional Sciences, University of Arizona, Tucson, Arizona, United States of America; 3 Department of Pediatrics, Division of Gastroenterology, Vanderbilt University School of Medicine, Nashville, Tennessee, United States of America; 4 Department of Biostatistics, Vanderbilt University Medical Center, Nashville, Tennessee, United States of America; 5 Department of Medicine, Department of Clinical Pharmacology, Vanderbilt University School of Medicine, Nashville, Tennessee, United States of America; Klinikum rechts der Isar der TU München, Germany

## Abstract

**Objectives:**

It is not established to what extent caloric intake must be reduced to lower oxidative stress in humans. The aim of this study was to determine the effect of short-term, moderate caloric restriction on markers of oxidative stress and inflammation in overweight and obese premenopausal women.

**Materials/Methods:**

Randomized trial comparison of 25% caloric restriction (CR) or control diet in 40 overweight or obese women (body mass index 32±5.8 kg/m^2^) observed for 28 days and followed for the next 90 days. Weight, anthropometry, validated markers of oxidative stress (F_2_-isoprostane) and inflammation (C-reactive protein), adipokines, hormones, lipids, interleukins, and blood pressure were assessed at baseline, during the intervention, and at follow-up.

**Results:**

Baseline median F_2_-isoprostane concentration (57.0, IQR = 40.5–79.5) in the CR group was 1.75-fold above average range for normal weight women (32.5 pg/ml). After starting of the caloric restriction diet, F_2_-isoprostane levels fell rapidly in the CR group, reaching statistical difference from the control group by day 5 (median 33.5, IQR = 26.0–48.0, P<0.001) and remained suppressed while continuing on the caloric restriction diet. Three months after resuming a habitual diet, concentrations of F_2_-isoprostane returned to baseline elevated levels in ∼80% of the women.

**Conclusions:**

Oxidative stress can be rapidly reduced and sustained through a modest reduction in caloric intake suggesting potential health benefits in overweight and obese women.

**Trial Registration:**

Clinicaltrials.gov NCT00808275

## Introduction

Obesity is associated with increased oxidative stress and chronic low-grade chronic inflammation [1]–[4]. Both events contribute to metabolic abnormalities occurring in the obesity-associated metabolic syndrome [1], [5]–[7] and play a critical role in the pathogenesis of various diseases such as atherosclerosis [8], [9], cancer [10], [11], cardiovascular disease [12], and diabetes type 2 [13]. Recent research has shown that weight loss attenuates inflammation and leads to improvement in adipokine profiles [14]–[16]. Although associations of overweight and obesity with increased oxidative stress have been reported, the effects of weight loss on oxidative stress markers are rarely described in literature [17]. Moreover, many clinical studies and intervention studies with diets or supplements have employed single measurements of F_2_-isoprostane before and after the intervention to estimate the oxidative stress without exploring intermittent or interval changes. To our knowledge, no prior study has investigated the association between caloric restriction (CR) and systemic short-term changes in markers of oxidative stress.

Thus, the goal of this controlled clinical trial was to determine whether modest (25%) short-term caloric restriction-induced weight loss affects systemic oxidative stress, as measured by changes in serum F_2_-isoprostane.

## Methods

**Figure 1:**
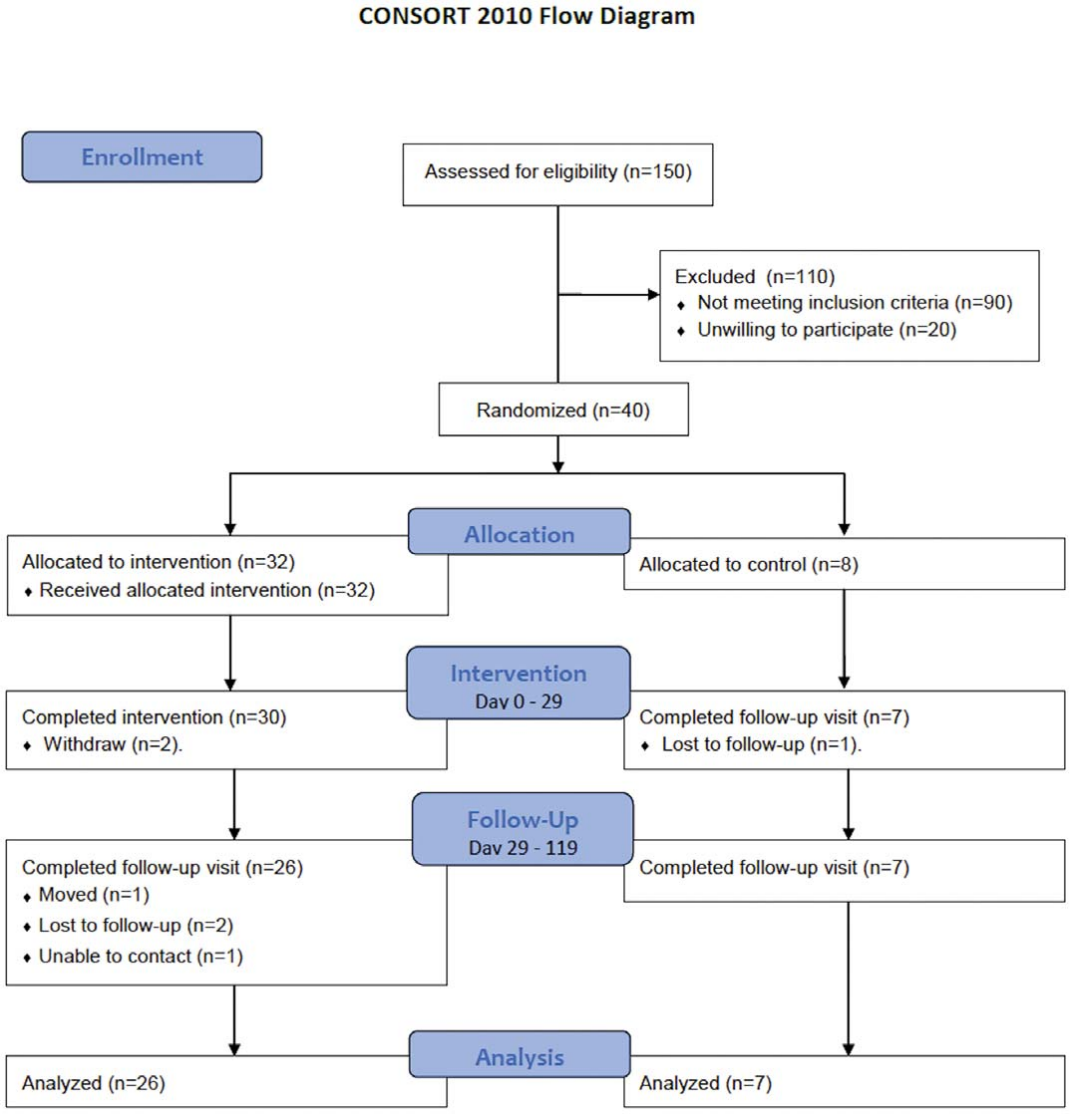
CONSORT flow diagram.

The protocol for this trial and supporting CONSORT checklist are available as supporting information; see Checklist S1 and Protocol S1. The protocol flow diagram is in Figure 1.

### Subjects

Forty premenopausal overweight and obese women, (18- to 45-years-old; body mass index - BMI: 25.4 to 43.0 kg/m^2^), were recruited from Nashville’s general population by advertisement in Vanderbilt University’s newspaper, mass emails, and flyers. Inclusion criteria were BMI more than 25 kg/m^2^ and willingness to abstain from alcohol consumption for the duration of the study, complete an overnight stay at the Clinical Research Center (CRC), and come to the CRC daily to obtain study foods. Volunteers were excluded if they reported having clinically significant illnesses (including type 2 diabetes), smokers, taking lipid-lowering medications, reported recent initiation/change in hormonal birth control or hormone replacement therapy, pregnant or lactating, taking medications or dietary supplements that affect body weight, or regularly engaging in vigorous physical activities. Participants classified their own ethnicity according to investigator-defined options. At a screening visit, all volunteers were measured for height, weight, and waist circumference, and completed a lifestyle questionnaire. Approximately 150 women were screened for eligibility based on the above criteria and 40 were enrolled. All applicable institutional and governmental regulations concerning the ethical use of human volunteers were followed during this research, in accordance with the ethical principles of the Helsinki-II Declaration. The trial (Clinicaltrials.gov Identifier: NCT00808275) was approved by the Institutional Review Board at Vanderbilt University and all participants provided written informed consent.

### Study Design

The study consisted of an experimental group that received a CR diet and a control group that remained on a habitual diet throughout the study. The ratio of women in CR group to the control group was 4 to 1 (allocated via blocked randomization by a statistician not involved in patient recruitment). The study was divided into a 7-day lead in period (Day -6 to Day 0), immediately followed by a 28-day intervention (Day 1 to Day 28), and a 3-month follow-up (Day 29 to Day 118). Dietary intervention in the CR group started during the luteal stage of the menstrual cycle (14–24 d) to control for confounding by menstrual cycle and any associated oxidative and/or inflammatory stress.

### Anthropometrics and Body Composition

The National Health and Nutrition Examination Survey (NHANES) protocols were followed for all anthropometrical measurements [18]. Body weight was measured at baseline and daily (for the experimental group) within 0.1 kg using a calibrated beam platform scale (Detecto-Medic, Detecto Scales, Inc, Northbrook, IL). Daily weight measurements were not revealed to participants and served as the criterion measure for dietary adherence. Height was measured at baseline within 0.5 cm using a calibrated wall-mounted stadiometer (Perspective Enterprises, Portage, MI). The average of two readings was used for analysis. All measurements were performed by the same investigator. Body composition (including fat mass and fat free mass) was measured at baseline (Day 0), during intervention (Days 14 and 28), and during the follow up visits (Days 42 and 119) using dual energy x-ray absorptiometry Lunar instrument (Lunar Prodigy, GE Medical Systems, Madison WI, adult software v. 9.15). For quality assurance and equilibration, a calibration block was scanned each morning and a spine phantom was scanned on a weekly basis (the coefficient of variation was 0.7%).

### Resting Energy Expenditure (REE)

REE was measured at Day 0 and 29 and was defined as the average EE during a 30-min period of lying in a supine position after a 30-min rest following an overnight fast (>10 h) using a whole-room indirect calorimeter. REE was calculated from measured rates of oxygen (O_2_) consumption and carbon dioxide (CO_2_) production using Weir’s equation [19]. The accuracy and precision of our metabolic chamber for measuring EE as determined by routine alcohol combustion test was 99.7% (IQR = 99.5−100.0%) over 24 hours and 98.5% (IQR = 96.9–99.9%) over 30 minutes.

### Dietary Intake

#### Caloric restriction diet

Participants in the CR group received individualized energy and nutrient controlled diets provided by the CRC metabolic kitchen for consumption at home. Energy needs were calculated as the sum of resting energy expenditure (REE) and energy expenditure of physical activity. Thermic effect of food was estimated as 10% of REE. Each individualized diet, provided in daily portions divided into 3 meals and 3 snacks, contained approximately 75% (±210 kcal) of daily energy requirements, including 52–62% of energy from carbohydrates, 25–29% of energy from fat, 18–23% of energy from protein, and 22–27 g of fiber. All participants received a multivitamin supplement daily (Nature Made, Mission Hills, CA). No restrictions were imposed on the amounts of energy-free foods ingested. Each participant received a written list of foods at daily pick-up. Any uneaten foods and any additional foods eaten by the participants were reported on sheets collected daily. The study dietitian met with each participant weekly to discuss the diet, resolve any barriers or concerns related to food or specimen collection, and encourage compliance. Energy and nutrient intake calculations were performed using the Nutrient Data System for Research (NDSR) software version 2009 developed by the Nutrition Coordinating Center, University of Minnesota, Minneapolis, MN, Food and Nutrient Database [20], [21]. Adherence to the protocol was monitored by urinary biomarkers (sodium, potassium, nitrogen) [22], [23].

#### Control diet

Participants in the control group were asked to follow their habitual diet for the study duration and they received a multivitamin supplement daily (Nature Made, Mission Hills, CA). Their dietary intake was assessed during the intervention (Days 1–28) from three 24-dietary recalls (2 weekdays and 1 weekend day) performed using automated multi-pass method and NDS-R software [24], [25].

### Physical Activity

Daily physical activity was assessed using an RT3 accelerometer (StayHealthy, Monrovia, CA, US). Participants were instructed to maintain their habitual physical activity level and wore an activity monitor on their right hip while awake for the duration of the intervention and twice for 7 days during the follow-up. Total and physical activity energy expenditure was calculated using energy calculated from the monitor-measured movement and measured REE. Physical activity levels (PAL) for each monitored day were calculated by dividing total energy expenditure by REE.

### Blood Pressure

Blood pressure was measured 3 times a week in the reclining position after 10 min rest with automatically inflating cuff (Dynamap, General Electric, Milwaukee, WI, USA) using a standard protocol.

### Blood Collection and Biochemical Measurements

Venous blood samples were drawn after an overnight fast at baseline and on days 1, 3, 5, 7, 14, 21, 29, 42, and 119, centrifuged immediately at 1 000 g for 10 minutes at 4°C, and the serum was stored in cryovials at –80°C until the assays were performed in batches. Samples obtained at baseline and during the study for each participant were included in the same assay run to avoid inter-assay variability among participants. F_2_-isoprostane was analyzed using gas chromatography/mass spectrometry (GC/MS), a previously described and validated method [26] with outstanding performance characteristics [27], [28]. F_2_-isoprostane assessing oxidation of lipids [29] has been shown to provide one of the most accurate assessments of oxidative stress status [27], [30]. Other methods (not used in this study) are targeting protein oxidation by measuring carbonyl groups in serum [31], DNA damage by measuring hydroxyl radical-induced products of DNA bases [32], and activity of several antioxidative enzymes including catalase, superoxide dismutase (SOD), glutathione S-transferase, and glutathione peroxidase (GPx) [33]–[35].

A basic metabolic panel, hematological indices (hemoglobin concentration, hematocrit, red and white blood cell counts), and C-reactive protein were analyzed in the Vanderbilt University Hospital Laboratory using standard methodologies. Blood for measurement of lipids, inflammatory cytokines, and hormones (leptin, insulin, adiponectin) was centrifuged, serum was extracted, and the samples were stored at −80°C until later analyses. Plasma triglyceride (TG), total cholesterol (TC), low-density lipoprotein (LDL), and high-density lipoprotein (HDL) levels were measured using enzymatic kits from Cliniqa Corporation (San Marcos, CA). Free fatty acids (FFA) were measured using the NEFA-C kit by Wako (Nneuss, Germany) and by gas chromatography. Glucose was measured using the Vitros Chemistry analyzer. Insulin and leptin measurements were performed using RIAs. Adiponectin was measured using a kit from Millipore (Billerica, MA) and Luminex multiplexing technology.

### Urine Collection and Analyses

Complete 24-h urine samples were collected once weekly. Urine volume and density was measured and a 10 mL sample was frozen at −70°C until further analysis. Urinary calcium, sodium, and potassium were measured using Vitros 250 Analyzer (Ortho-Clinical Diagnostics, Rochester, NY, USA). Urinary nitrogen content was measured using nitrogen analyzer (Antek Instrument Nitrogen System 9000NS, Antek Instruments, Inc., Houston, TX, USA). The nitrogen excretion in the urine was used as a biological marker for protein intake by multiplying the content of nitrogen in the urine by the factor 7.72 [22]. The urine sodium and potassium contents were used as biological markers of sodium and potassium intake, respectively. Urinary creatinine was measured on a Sirrus Clinical Chemistry analyzer (Stanbio Laboratory, Boerne, TX).

### Statistical Analysis

Descriptive statistics were presented as mean and standard deviation (SD) or median and IQR or percentage, as appropriate. Daily caloric intake and REE were expressed as the absolute and deficit number of kilocalories per day. Continuous endpoints were compared between the control and intervention group using Wilcoxon rank sum test. The change within group was assessed using Wilcoxon signed rank test. Spearman correlation coefficient was used to assess the correlation between two continuous variables. Multivariable linear model was used to assess the treatment effect at single time point while adjusting for the baseline measures. We performed a linear model using generalized least squares with autocorrelation structure of order 1 (AR1) for the within-subject correlation to assess the F_2_-isoprostane change within 28 days of study period. The main effects included baseline age, BMI, and F_2_-isoprostane, time, group, and time by group interaction. Time was modeled as nonlinear relationship to the F_2_-isoprostane using restricted cubic splines. Residual plot and quantile-quantile (QQ) plots were used to check the model assumptions. The level of statistical significance was set at p<0.05. All analyses were done with STATA 11 (StataCorp, College Station, TX) and the statistical programming language R, version 2.13.1 (R Development Core Team, Vienna, Austria).

## Results

### Baseline Characteristics and Compliance

Thirty of 32 participants in the CR group and all controls completed the intervention part of the study (i.e., returned for the Day 29 visit). The reasons for study dropout were work related (n = 1) and a family emergency (n = 1). Twenty-six CR and 7 control group participants completed the entire study. The reasons for study dropout were work-related (CR, n = 2; control, n = 1), pregnancy (CR, n = 1), and an unspecified reason (CR, n = 1). The participants who did not complete the CR protocol did not differ from the completers in regards to age, body weight, body fat, CRP, or insulin, but did have lower baseline F_2_-isoprostane plasma concentrations (38.5, IQR = 20.5–48.2 vs. 63.5, IQR = 45.8–79.0; P = 0.01). Baseline demographic and anthropometric characteristics are shown in Table 1. Adherence to the protocol in completers was good as measured by urinary biomarkers calculated as ratios of reported intake and excretion for protein (1.03±0.25), sodium (0.95±0.19) and potassium (1.04±0.32) (Table 2).

**Table 1:** Baseline demographic and anthropometric characteristics.

	Control diet (n = 8)	CR diet (n = 32)
Age (years)	29.5 (26.8–33.3)	30.5 (26.0–34.0)
Body weight (kg)	86.9 (72.2–96.2)	85.2 (70.4–99.4)
Body-mass index (kg/m^2^)	30.1 (28.1–38.3)	32.3 (26.6–36.3)
Body fat mass (kg)	39.3 (33.2–44.1)	38.6 (27.5–49.0)
*Ethnicity*		
African American	3	9
Caucasian	4	22
Other (Asian, Hispanic)	1	1

Data are presented as median (interquartile range - IQR).

Abbreviations: CR diet, caloric restriction diet.

**Table 2:** Comparison of reported average (28 days) intake of protein, sodium, and potassium with the corresponding biological markers in urine in the caloric restriction (CR) diet group.

	Intake^a^	Urinary Excretion^b^	Ratio of Intake to Excretion	p-value^c^
Protein (g/day)	85.0±12.0	87.9±3.6	1.03±0.25	0.486
Sodium (g/day)	3.03±0.44	3.18±0.83	0.95±0.19	0.919
Potassium (g/day)	2.93±0.43	2.83±0.43	1.04±0.32	0.553

Data are presented as means ± s.d.

a Average daily intakes of protein, sodium, and potassium assessed by NDSR (Nutrition Diet System, St. Paul, MN).

b Excretion measured from 4 weekly 24-hour urine collections. Protein = urinary nitrogen *7.72 (18), Potassium = urinary potassium *0.77 (19).

c Comparison of ratios of intake to excretion; one sample t-test.

### Changes in F_2_-isoprostane Concentrations

**Figure 2:**
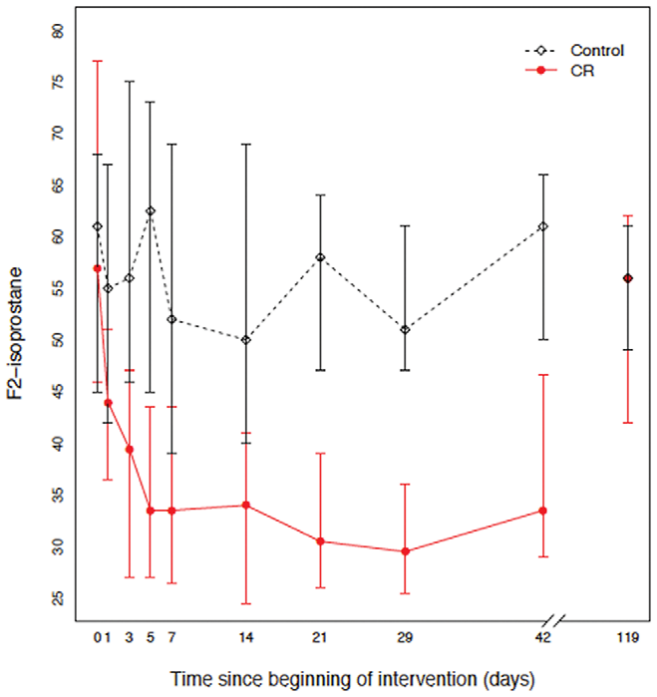
Median plasma concentrations of F_2_-isoprostane (prostaglandin F_2_-like compound) in overweight and obese women at baseline (Day 0), during dietary intervention (Days 1–29), and after 90-day follow-up (Days 42 and 119) in the CR (caloric restricted) diet (75% of energy requirement) group (n = 32) and in the control group (n = 8) without diet restrictions (100% of energy requirement). Vertical bars represent 95% bootstrap confidence intervals.

We fit a linear model of F_2_-isoprostane within the 28 days study period using generalized least squares. F_2_-isoprostane was log transformed since the distribution was skewed. There were statistically significant differences between the CR and control group (P<0.001). The biggest difference appeared at day 14 (control/CR, 1.79, 95% CI: 1.50–2.13) and day 21 (control/CR, 1.82, 95% CI: 1.56–2.11). (Figure 2, Table 3). In participants with BMI>30 (n = 14), plasma F_2_-isoprostane concentrations decreased by more than 50% during the first week (Day 1 to Day 7). After the 3-month follow-up period, plasma F_2_-isoprostane concentrations in the CR group increased and the difference between the groups was not significant (P = 0.692) (Table 4).

**Table 3:** Multivariable linear regression results for change in F2-isoprostane serum levels during the 28-day dietary intervention.

Factor	Effect	95% CI	p-value
Baseline age [years] - 34∶26	0.95	0.89–1.02	0.150
Baseline BMI [kg/m^2^] - 36.4∶26.9	1.12	1.02–1.21	0.015
Baseline F_2_-isoprostane (pg/mL) - 78.2∶44.8	1.46	1.36–1.55	<0.001
Day of intervention - day 21:day 3	0.76	0.69–0.84	<0.001
Group - Control : CR	1.60	1.36–1.88	<0.001

Effect shown is the F_2_-isoprostane ratio. BMI - body mass index; CR diet- caloric restriction diet. Adjusted to: Day of intervention = 7, Group = CR.

**Table 4:** Differences in F_2_-isoprostane (pg/mL) serum levels between caloric restriction (CR) and control groups at baseline (Day 0), during the intervention (Days 1 to 29), and follow-up (Days 42 and 119).

Time	N	Control diet	CR diet	p-value^a^
Baseline	40	61.0 (51.8–65.0)	57.0 (40.5–79.5)	0.908
Day 1	40	55.0 (48.8–65.0)	44.0 (35.5–61.2)	0.153
Day 3	40	56.0 (46.8–73.5)	39.5 (24.8–50.2)	0.010
Day 5	40	62.5 (45.0–72.5)	33.5 (26.0–48.0)	0.002
Day 7	40	52.0 (45.0–66.0)	33.5 (23.8–45.8)	0.007
Day 14	40	50.0 (42.0–67.5)	34.0 (23.5–43.5)	0.004
Day 21	40	58.0 (49.2–60.2)	30.5 (23.8–42.5)	<0.001
Day 29	38	51.0 (49.2–53.5)	29.5 (23.2–40.8)	<0.001
Day 42	37	61.0 (55.0–65.0)	33.5 (29.0–53.5)	0.006
Day 119	34	56.0 (52.0–59.5)	56.0 (36.0–66.0)	0.692

Data are presented as median (interquartile range- IQR). CR diet, caloric restriction diet.

a Wilcoxon rank sum test for differences between Control and CR diets.

### Energy Balance and Weight Changes

The average caloric intake was significantly lower in the CR than in the control group with an average intake of 17.8±2.2 kcal/kg/day vs. 28.2±4.6 kcal/kg/day (P<0.001). By the end of the dietary intervention (Day 29), total body weight and body fat weight decreased in the CR more than in the control group (difference 2.7 kg and 1.1 kg, respectively). However, differences in body weight and body fat between CR and control groups were non-significant at baseline, end of intervention, and day 119 follow-up (Table 5). There was no significant between group difference in REE adjusted for fat free mass. The total amount of physical activity-related EE (kcal/day) was lower in the CR than the control group at Day 29 (difference of 61.2 kcal/day, P = 0.057). However, there was no significant between group difference (P = 0.303) in physical activity level (PAL) at the end of the intervention period (Table 6). Median intake of energy and macronutrients was lower in the CR than in control group (P<0.001), as expected (Table 7).

**Table 5:** Body weight, BMI, and body fat mass at baseline, end of intervention (Day 29), and follow-up (Day 119).

Variable	Time	Control diet	CR diet	p-value^a^
Body Weight(kg)	Baseline	86.9 (72.2–96.2)	85.5 (70.4–99.4)	0.740
	Day 29	86.9 (71.1–97.6)	82.8 (68.5–95.8)	0.504
	Day 119	87.8 (79.7–98.7)	84.5 (72.6–96.3)	0.491
BMI (kg/m^2^)	Baseline	30.1 (28.1–38.3)	32.3 (26.6–36.2)	0.538
	Day 29	30.1 (27.6–38.8)	31.5 (25.7–35.3)	0.438
	Day 119	30.0 (28.5–39.0)	32.1 (26.2–35.8)	0.329
Body Fat (kg)	Baseline	39.3 (33.2–44.1)	38.5 (27.4–49.0)	0.946
	Day 29	39.9 (31.0–44.0)	38.0 (26.0–47.0)	0.598
	Day 119	39.1 (33.5–45.9)	36.7 (27.8–44.3)	0.544

Data are presented as median (interquartile range - IQR).

Abbreviations: CR diet, caloric restriction diet; BMI, body mass index (kg/m^2^).

a Wilcoxon rank sum test for differences between Control and CR diets.

**Table 6:** Resting energy expenditure, physical activity-related energy expenditure and physical activity level (PAL) at baseline, end of intervention (Day 29), and day follow-up (Day 119).

Variable	Time	Control diet	CR diet		p-value^a^
REE	Baseline	1.33 (1.22–1.37)		1.22 (1.18–1.37)	0.255
(kcal/min)	Day 29	1.33 (1.21–1.38)		1.20 (1.17–1.35)	0.191
	Day 119	1.35 (1.27–1.39)		1.28 (1.19–1.40)	0.330
PAEE	Baseline	502.7 (471.7–514.3)		519.5 (423.3–542.5)	0.740
(kcal/day)	Day 29	554.0 (511.0–648.1)		492.8 (463.1–553.9)	0.057
	Day 119	505.5 (476.8–540.3)		497.8 (425.4–514.5)	0.415
PAL	Baseline	1.31 (1.31–1.32)		1.27 (1.25–1.32)	0.078
	Day 29	1.33 (1.31–1.33)		1.29 (1.25–1.35)	0.303
	Day 119	1.32 (1.31–1.33)		1.27 (1.25–1.31)	0.012

Data are presented as median (interquartile range -IQR).

Abbreviations: REE, resting energy expenditure; PAEE, physical activity-related energy expenditure calculated as a difference between total and resting energy expenditure; PAL, physical activity level calculated as a ratio of total energy expenditure to REE, CR diet, caloric restriction diet.

a Wilcoxon rank sum test for differences between Control and CR diets.

**Table 7:** Median daily macro- and selected micro-nutrient intake in the caloric restriction (CR) and Control diet groups.

	Control diet				CR diet		
Nutrient	Median	IQR		Median			IQR
Energy (kcal)	2062.4	(1906.1–2119.9)	1543.3^1^			(1410.9–1784.1)	
Fat (g)	54.7	(52.8–67.6)	44.2^1^			(33.5–54.6)	
Carbohydrate (g)	276.1	(253.5–329.6)	227.9^1^			(213.1–253.9)	
Protein (g)	101.2	(93.3–124.2)	82.0^1^			(70.9–93.4)	
Glycemic Load (bread reference)	213.7	(197.3–241.3)	162.1^1^			(141.5–183.4)	
Dietary Fiber (g)	21.9	(18.8–24.3)	24.4			(22.1–27.3)	
Vitamin A (retinol equivalents) (mcg)	1886.8	(1736.8–2107.2)	2034.7			(2202.3–6755.1)	
Vitamin D (IU)	337.4	(293.4–392.8)	325.2			(312.6–355.0)	
Vitamin E (IU)	8.5	(7.1–12.0)	7.7			(6.4–9.3)	
Vitamin C (ascorbic acid) (mg)	87.0	(73.0–116.3)	127.1			(100.9–167.1)	
Vitamin B_1_ (thiamin) (mg)	1.5	(1.4–1.7)	1.2			(1.0–1.6)	
Vitamin B_2_ (riboflavin) (mg)	2.2	(2.1–2.8)	1.9			(1.7–2.1)	
Vitamin B_6_ (mg)(piridoxal, piridoxamine, piridoxin)	2.0	(1.8–2.1)	1.5			(1.3–1.8)	
Niacin (mg)	22.5	(20.9–24.9)	19.4			(15.0–22.5)	
Folate (mcg)	574.3	(524.8–685.5)	501.7			(464.5–552.4)	
Vitamin B_12_ (cobalamin) (mcg)	6.9	(5.7–7.6)	4.6			(3.4–6.1)	
Sodium (mg)	3588.8	(3319.3–4209.7)	2604.4			(2112.0–3433.0)	
Calcium (mg)	822.3	(729.1–920.5)	1008.6			(881.6–1147.2)	
Magnesium (mg)	509.2	(451.7–540.7)	455.3			(436.8–482.1)	
Iron (mg)	14.4	(12.1–15.2)	9.4			(7.7–11.5)	

Data are presented as median (interquartile range -IQR).

1 - different from Control diet, p<0.001, Wilcoxon rank sum test.

Daily multivitamin supplement was given during intervention Days 1–28 (Multi Complete, Nature Made, Mission Hills, CA) composition: vitamin A - 2 500 IU, vitamin D_3_ - 1000 IU, vitamin E - 50 IU, vitamin C - 180 mg, vitamin B_1_ - 15 mg, vitamin B_2_ - 1.7 mg, niacin - 20 mg, vitamin B_6_ - 2 mg, folate - 400 mcg, vitamin B_12_ - 6 mcg, calcium 162 mg, magnesium - 100 mg, iron - 18 mg, selenium - 70 mcg.

### Changes in other Disease Associated Markers

The CR diet did not have a significant effect on either systolic or diastolic blood pressure or serum concentrations of insulin, leptin, adiponectin, total cholesterol, LDL cholesterol, HDL cholesterol, CRP and triglycerides (Tables 8 and 9).

**Table 8:** Blood pressure, hormones, and lipids at baseline, end of intervention (Day 29), and follow-up (Day 119).

Variable	Time	Control diet	CR diet	p-value^1^
Systolic blood	Baseline	121.5 (120.1–126.5)	128.5 (122.8–132.3)	0.052
pressure (mmHg)	Day 29	125.0 (123.0–126.0)	123.0 (117.3–129.0)	0.648
	Day 119	126.0 (124.5–128.0)	125.0 (121.5–131.5)	0.897
Diastolic blood	Baseline	79.5 (76.5–82.5)	77.5 (74.0–82.0)	0.206
pressure (mmHg)	Day 29	79.0 (78.0–81.5)	77.0 (73.0–81.0)	0.116
	Day 119	77.0 (75.0–80.0)	77.0 (75.0–79.8)	0.983
Insulin (uU/mL)	Baseline	13.8 (7.0–16.8)	11.7 (8.9–17.0)	0.803
	Day 29	11.1 (8.1–17.0)	9.8 (6.8–12.6)	0.514
	Day 119	11.5 (7.5–16.1)	10.1 (8.9–15.2)	0.830
Leptin (ng/mL)	Baseline	19.5 (16.5–25.6)	21.1 (15.4–28.0)	0.974
	Day 29	19.6 (16.4–31.1)	17.8 (11.6–24.2)	0.250
	Day 119	17.8 (15.9–25.9)	22.6 (15.4–26.8)	0.784
Adiponectin	Baseline	9.1 (5.5–10.2)	10.0 (7.7–13.8)	0.235
(ng/mL)	Day 29	7.2 (5.9–11.3)	12.0 (7.8–15.7)	0.114
	Day 119	8.1 (7.0–9.9)	12.1 (8.7–17.7)	0.243
Triglyceride	Baseline	134.5 (131.5–154.0)	138.0 (122.0–153.2)	0.595
(mg/dL)	Day 29	142.0 (131.0–157.0)	132.0 (118.0–144.0)	0.125
	Day 119	126.0 (124.5–149.0)	135.0 (121.5–145.0)	0.587
Totalcholesterol	Baseline	214.5 (189.5–241.2)	208.0 (181.0–226.2)	0.454
(mg/dL)	Day 29	214.5 (188.8–227.8)	190.5 (174.2–210.8)	0.378
	Day 119	215.0 (205.0–231.5)	209.0 (186.0–220.0)	0.391
LDL (mg/dL)	Baseline	148.8 (110.0–167.8)	132.0 (105.8–150.2)	0.377
	Day 29	152.0 (122.2–159.2)	118.5 (107.2–143.8)	0.208
	Day 119	149.0 (137.5–153.5)	125.0 (117.0–142.5)	0.132
HDL(mg/dL)	Baseline	45.5 (36.0–53.5)	46.0 (42.0–54.2)	0.752
	Day 29	42.0 (35.0–46.0)	44.0 (35.0–54.0)	0.515
	Day 119	48.0 (40.5–50.5)	46.0 (41.0–52.0)	0.573
Free fatty acids	Baseline	0.41 (0.32–0.60)	0.53 (0.37–0.73)	0.262
(mmol/L)	Day 29	0.41 (0.31–0.53)	0.52 (0.42–0.67)	0.108
	Day 119	0.38 (0.35–0.55)	0.49 (0.40–0.65)	0.156

Data are presented as median (interquartile range -IQR).

Abbreviations: LDL, low-density lipoprotein; HDL, high-density lipoprotein.

1 Wilcoxon rank sum test for differences between Control and CR diets.

**Table 9:** Inflammatory markers at baseline, end of intervention (Day 29), and follow-up (Day 119).

Variable	Time	Control diet	CR diet	p-value^1^
Tumor necrosis	Baseline	5.67 (1.65–9.25)	2.88 (0.00–5.22)	0.310
factor -á (ug/dL)	Day 29	5.90 (1.40–9.11)	3.08 (0.00–8.00)	0.555
	Day 119	7.67 (2.48–8.81)	4.96 (0.00–5.68)	0.340
Interleukin -12	Baseline	6.47 (5.30–9.52)	2.75 (0.00–5.68)	0.027
(pg/mL)	Day 29	9.53 (5.70–10.39)	3.19 (0.00–5.81)	0.027
	Day 119	8.33 (7.48–9.39)	4.26 (0.00–6.94)	0.007
Interleukin -8	Baseline	8.55 (8.20–17.73)	5.02 (3.04–8.37)	0.003
(pg/mL)	Day 29	9.72 (7.32–39.30)	6.48 (4.87–8.29)	0.019
	Day 119	7.41 (7.16–25.88)	5.94 (2.65–8.55)	0.075
C-reactiveprotein	Baseline	6.00 (3.00–9.50)	3.50 (1.00–6.00)	0.157
(ug/mL)	Day 29	4.20 (2.65–7.25)	2.60 (1.00–6.25)	0.274
	Day 119	4.70 (1.12–9.10)	3.00 (1.00–5.07)	0.776

Data are presented as median (interquartile range - IQR). CR, caloric restriction diet.

1 Wilcoxon rank sum test for differences between Control and CR diets.

## Discussion

The novelty of the present study is that we investigated serial changes in a marker of oxidative stress induced by 28-days of a caloric restriction diet. The main finding is that moderate (25%) CR and modest weight loss causes a rapid decrease in oxidative stress as measured by plasma F_2_-isoprostane concentrations, to within the normal range exhibited by non-obese adults (35±6 pg/ml). The magnitude of this change was significant in comparison to baseline F_2_-isoprostane plasma concentrations. This suggests that the potential benefits from reducing the oxidative stress level can be achieved rapidly without restricting caloric intake to a level that overweight and obese people might find difficult to sustain.

CR is hypothesized to lessen oxidative damage by reducing energy flux and metabolism with a consequential lowering of reactive oxygen species and rate of oxidative damage to vital tissues [36]. Several studies documented the association of CR with lowering of resting metabolic rate and thermic effect of food, and a decrease in cost of physical activity [37]. For example, Heilbronn et al [38] showed 6 months of caloric restriction (25%) significantly decreased sedentary 24-hour energy expenditure after adjusting for body composition. In the present study, we did not observe significant reductions in adjusted energy expenditure most likely because of a much shorter intervention period (1 month vs. 6 months). Other reported benefits of long-term CR, but not detected in our study, include reduction in fasting glucose and insulin, which are linked to decreased insulin resistance and risk for type 2 diabetes [39].

Our results showing decreased F_2_-isoprostane with weight loss are consistent with a previous Davi et al [40] study involving 11 obese women who participated in a diet-induced weight loss program for a 12-week period and lost at least 5 kg of initial body weight. In eight of these women, there was a significant reduction in urinary excretion of F_2_-isoprostane. In a recent study on the effect of CR and glycemic load on oxidative stress, Meydani et al [17] showed non-significant decreases in plasma 8-epi-prostaglandin F_2á_ (P = 0.09) and protein carbonyl (P = 0.02), and a significant increases in plasma glutathione peroxidase activity (P = 0.04). In contrast, a randomized controlled trial with 42 participants with the metabolic syndrome assigned to 16 weeks of weight maintenance or a 12-week weight-loss program followed by 4 weeks of weight stabilization, showed different results. Relative to the weight-maintenance group, a 4-kg loss in weight resulted in a significant decrease in blood pressure but did not alter urinary or plasma F_2_-isoprostane [41]. The discrepancies between these studies could be explained, at least in part, by different methodology (plasma or urinary isoprostanes), various experimental designs, and diverse study populations.

Baseline F_2_-isoprostane correlated with body fat content. However, the F_2_-isoprostane decrease during the CR intervention was not correlated with the concurrent changes in body fat. Although we used a clinical trial design, strict diet control, and validated biomarkers [42], [43], it was not possible to determine whether body fat itself was a source of oxidative stress. As far as we are aware, F_2_-isoprostanes, although present in foods, are not absorbed through the gut [44], [45]. A plausible explanation is that in addition to a decrease in energy content, changes in content and amount of macronutrients consumed affected systemic oxidative balance. Lower concentrations of protein, carbohydrates, and lipids in the CR diet, when compared to habitual diets reported by Control diet participants, could have potentially shifted postprandial oxidative status towards decreased susceptibility to oxidative damage [46]. Indeed, previous research has shown that postprandial increases of lipid and carbohydrate concentrations lead to increased oxidative stress [47] and consequent hyperlipidemia and hyperglycemia [48].

Although our study did not test whether decreases in oxidative stress are linked to an improvement in risk factors for associated chronic diseases, previous data do provide such evidence. Zacardi et al [49] reported that declines in adhesion molecules and improvement in endothelial function with sustained weight loss were related to decreases in IL-6 and TNF-α, independent of change in adiposity and body fat distribution. Despite the many studies that have examined the role of oxidative stress on cardiovascular health, mechanistic studies designed to abruptly reduce plasma oxidative stress and to subsequently determine the acute effects of this intervention on antioxidative capacity, oxidative stress, and vascular function have not been extensively reported. For example, studies in young and older individuals demonstrated a dichotomous effect of antioxidant consumption on endothelial function with age [50]. Nevertheless, if the link between obesity and increased oxidative stress is confirmed, the potential for antioxidant therapy to decrease the risk of obesity-related co-morbid conditions such as cardiovascular disease warrants additional studies in diverse populations [51].

Our findings extend those of previous studies in several ways. First, our data provide evidence from a controlled trial design that the decrease in oxidative stress biomarkers induced by a modest caloric restriction is rapid. This is important because obese individuals are unable to consistently comply with a long-term daily caloric reduction of 40% (consuming 60% of maintenance), as has been used in most animals studies [52]. Second, the study causally links the measured decreases in F_2_-isoprostane to the weight loss induced by caloric restriction. Third, we demonstrated that return to a habitual diet, with consequent gradual regaining of weight, causes an increase of F_2_-isoprostane to pre-study, elevated baseline levels. This observation may have clinical significance especially in weight cycling (yo-yo dieting) [53], [54] and weight-loss maintenance [55]. It has been reported that weight cycling is a relatively common phenomenon in women, ranging from 19% in White [56], [57] to 63% in African American women [58], as well as in approximately 10% of men [57]. Weight cycling has a negative impact on body composition and body fat distribution [59], [60], and has been associated with an increased risk for metabolic syndrome [61],cardiovascular disease [62], and all-cause mortality [63]. Now we have a clear understanding of how weight cycling stimulates adjustments in energy homeostatic hormones (leptin, ghrelin, and insulin) that activate regulatory mechanisms to restore weight [64].

However, we do not know whether energy intake-induced weight cycling is affecting oxidative stress level. Our results support the notions that during caloric restriction and subsequent weight loss, oxidative stress level decreases, and in contrast, increases during a positive caloric balance and subsequent weight gain. Thus, we hypothesize that previously mentioned health consequences of weight cycling such as increased risk for metabolic syndrome and cardiovascular disease could be associated, at least in part, with changes in the oxidative stress level. Future clinical studies are necessary to explain whether these associations can be attenuated by frequent weight cycling and what mechanisms (e.g. lipids oxidation, insulin resistance, and inflammation) are involved.

A limitation of the present study is the relatively small number of participants; hence, the results need to be confirmed in larger studies. In addition, individuals in the control group ate their habitual diets, thus it was possible that their intake of antioxidants (e.g., vitamin E,) and other micronutrient would be below the recommended allowances. To prevent this possibility, participants in both groups received a vitamin/mineral supplement daily. As a result, vitamin E intake was similar in the control and CR groups (∼58 international units), which was in line with the daily recommended intake of 22.4 to 50 international units for younger and older adults, respectively [65].

Although the use of F_2_-isoprostanes as a marker of oxidative stress is a strength [66], the study would have greatly benefited from measurement of water-soluble oxidation markers such as thiobarbituric acid reactive substances [39] and total antioxidant capacity [67], [68]. However, in the study on effect of diet-induced reduction in oxidative stress it has been showed that F_2_-isoprostane level was well correlated with advanced oxidation protein products [69]. In addition, one should be cautious if generalizing this study’s finding to males, obese individuals, and populations with serious obesity-related diseases, such as type 2 diabetes or hyperlipidemias. Further studies will be required to determine whether caloric restriction and weight loss lead to a reduction in F_2_-isoprostane and other markers of oxidative stress in such conditions. Finally, we did not explore the effect of age on our results. For example, it has been shown that antioxidant consumption acutely restores endothelial function in the elderly while disrupting normal endothelium-dependent vasodilation in the young, and suggest that this age-related impairment is attributed, at least in part, to free radicals [70]. However, participants in our study were relatively healthy premenopausal women eliminating, at least in part, the potential effect of age on the results.

In summary, the results of the present study show that oxidative stress can be rapidly reduced and sustained through a modest (25%) reduction in caloric intake for a relatively short (28-day) period. Simultaneous reduction in markers of inflammation was associated with decreases in body fat and body weight. These changes suggest potential health benefits of modest caloric restriction in overweight and obese women.

## Supporting Information

Checklist S1 **CONSORT Checklist.**
(DOC)

Protocol S1 **Trial Protocol.**
(DOCX)

## References

[1] Vincent HK, Innes KE, Vincent KR (2007) Oxidative stress and potential interventions to reduce oxidative stress in overweight and obesity. Diabetes, Obesity and Metabolism 9: 813–839. 10.1111/j.1463-1326.2007.00692.x 17924865

[2] Lumeng CN, Saltiel AR (2011) Inflammatory links between obesity and metabolic disease. The Journal of Clinical Investigation 121: 2111–2117. 21633179 10.1172/JCI57132 PMC3104776

[3] Hotamisligil GS (2006) Inflammation and metabolic disorders. Nature 444: 860–867. 17167474 10.1038/nature05485

[4] Basu S (2008) F2-Isoprostanes in Human Health and Diseases: From Molecular Mechanisms to Clinical Implications. Antioxidants & Redox Signaling 10: 1405–1434. 18522490 10.1089/ars.2007.1956

[5] Furukawa S, Fujita T, Shimabukuro M, Iwaki M, Yamada Y, et al (2004) Increased oxidative stress in obesity and its impact on metabolic syndrome. The Journal of Clinical Investigation 114: 1752–1761. 15599400 10.1172/JCI21625 PMC535065

[6] Tinahones FJ, Murri-Pierri M, Garrido-Sanchez L, Garcia-Almeida JM, Garcia-Serrano S, et al (2008) Oxidative Stress in Severely Obese Persons Is Greater in Those With Insulin Resistance. Obesity 17: 240–246. 19023278 10.1038/oby.2008.536

[7] Ouchi N, Parker JL, Lugus JJ, Walsh K (2011) Adipokines in inflammation and metabolic disease. Nat Rev Immunol 11: 85–97. 21252989 10.1038/nri2921 PMC3518031

[8] Keaney JF Jr, Larson MG, Vasan RS, Wilson PWF, Lipinska I, et al (2003) Obesity and Systemic Oxidative Stress: Clinical Correlates of Oxidative Stress in The Framingham Study. Arterioscler Thromb Vasc Biol 23: 434–439. 12615693 10.1161/01.ATV.0000058402.34138.11

[9] Sawyer DB (2011) Oxidative Stress in Heart Failure: What Are We Missing? The American Journal of the Medical Sciences 342: 120–124 doi: 110.1097/MAJ.1090b1013e3182249fcd. 21747279 10.1097/MAJ.0b013e3182249fcd PMC3145812

[10] Barocas DA, Motley S, Cookson MS, Chang SS, Penson DF, et al (2011) Oxidative Stress Measured by Urine F2-Isoprostane Level is Associated With Prostate Cancer. The Journal of Urology 185: 2102–2107. 21496850 10.1016/j.juro.2011.02.020 PMC3093434

[11] De Marzo AM, Platz EA, Sutcliffe S, Xu J, Gronberg H, et al (2007) Inflammation in prostate carcinogenesis. Nat Rev Cancer 7: 256–269. 17384581 10.1038/nrc2090 PMC3552388

[12] Couillard C, Ruel G, Archer WR, Pomerleau S, Bergeron J, et al (2005) Circulating levels of oxidative stress markers and endothelial adhesion molecules in men with abdominal obesity. Journal of Clinical Endocrinology & Metabolism 90: 6454–6459. 16189262 10.1210/jc.2004-2438

[13] Helmersson J, Vessby B, Larsson A, Basu S (2004) Association of type 2 diabetes with cyclooxygenase-mediated inflammation and oxidative stress in an elderly population. Circulation 109: 1729–1734. 15037525 10.1161/01.CIR.0000124718.99562.91

[14] Tchernof A, Nolan A, Sites CK, Ades PA, Poehlman ET (2002) Weight Loss Reduces C-Reactive Protein Levels in Obese Postmenopausal Women. Circulation 105: 564–569. 11827920 10.1161/hc0502.103331

[15] Klimcakova E, Kovacikova M, Stich V, Langin D (2010) Adipokines and dietary interventions in human obesity. Obesity Reviews 11: 446–456. 20059706 10.1111/j.1467-789X.2009.00704.x

[16] Rolland C, Hession M (2011) Broom (2011) Effect of weight loss on adipokine levels in obese patients. Diabetes Metab Syndr Obes 4: 315–323. 21887104 10.2147/DMSO.S22788 PMC3160856

[17] Meydani M, Das S, Band M, Epstein S, Roberts S (2011) The effect of caloric restriction and glycemic load on measures of oxidative stress and antioxidants in humans: results from the CALERIE Trial of Human Caloric Restriction. J Nutr Health Aging 15: 456–460. 21623467 10.1007/s12603-011-0002-z PMC3229089

[18] National Health and Nutrition Examination Survey (NHANES): Anthropometry Procedures Manual. In: Prevention CfDCa, editor. Atlanta, GA: CDC. 3: 20–23 and 23: 30–21.

[19] Weir J (1949) New methods for calculating metabolic rate with special reference to protein metabolism. The Journal of Physiology 109: 1–9. 15394301 10.1113/jphysiol.1949.sp004363 PMC1392602

[20] Miller PE, Mitchell DC, Harala PL, Pettit JM, Smiciklas-Wright H, et al (2011) Development and evaluation of a method for calculating the Healthy Eating Index-2005 using the Nutrition Data System for Research. Public Health Nutrition 14: 306–313. 20576195 10.1017/S1368980010001655

[21] Harnack L, Stevens M, Van H, Schakel S, Dwyer J, et al. (2008) A computer-based approach for assessing dietary supplement use in conjunction with dietary recalls. J Food Compost Anal 21 (Supplement): S78–S82. 10.1016/j.jfca.2007.05.004 PMC2151738 19190705

[22] Bingham S, Cummings J (1985) Urine nitrogen as an independent validatory measure of dietary intake: a study of nitrogen balance in individuals consuming their normal diet. The American Journal of Clinical Nutrition 42: 1276–1289. 4072961 10.1093/ajcn/42.6.1276

[23] McCullough ML, Swain JF, Malarick C, Moore TJ (1991) Feasibility of outpatient electrolyte balance studies. Journal of the American College of Nutrition 10: 140–148. 2030256 10.1080/07315724.1991.10718138

[24] Feskanich D, Sielaff BH, Chong K, Buzzard IM (1989) Computerized collection and analysis of dietary intake information. Comput Methods Programs Biomed 30: 47–57. 2582746 10.1016/0169-2607(89)90122-3

[25] Johnson RK, Driscoll P, Goran MI (1996) Comparison of multiple-pass 24-hour recall estimates of energy intake with total energy expenditure determined by the doubly labeled water method in young children. J Am Diet Assoc 96: 1140–1144. 8906138 10.1016/S0002-8223(96)00293-3

[26] Morrow JD, Roberts LJ 2nd (1999) Mass spectrometric quantification of F2-isoprostanes in biological fluids and tissues as measure of oxidant stress. Methods in enzymology 300: 3–12. 9919502 10.1016/s0076-6879(99)00106-8

[27] Kadiiska MB, Gladen BC, Baird DD, Germolec D, Graham LB, et al (2005) Biomarkers of Oxidative Stress Study II: Are oxidation products of lipids, proteins, and DNA markers of CCl4 poisoning? Free Radical Biology and Medicine 38: 698–710. 15721980 10.1016/j.freeradbiomed.2004.09.017

[28] Milne GL, Musiek ES, Morrow JD (2005) F2-Isoprostanes as markers of oxidative stress in vivo: An overview. Biomarkers 10: 10–23. 16298907 10.1080/13547500500216546

[29] Morrow JD, Hill KE, Burk RF, Nammour TM, Badr KF, et al (1990) A Series of Prostaglandin F2-Like Compounds are Produced in vivo in Humans by a Non-Cyclooxygenase, Free Radical-Catalyzed Mechanism. PNAS 87: 9383–9387. 2123555 10.1073/pnas.87.23.9383 PMC55169

[30] Milne GL, Yin H, Hardy KD, Davies SS, Roberts LJ (2011) Isoprostane Generation and Function. Chemical Reviews 111: 5973–5996. 21848345 10.1021/cr200160h PMC3192249

[31] Chevion M, Berenshtein E, Stadtman ER (2000) Human studies related to protein oxidation: protein carbonyl content as a marker of damage. Free Radic Res 33 Suppl: S99–108 11191280

[32] Dizdaroglu M, Nackerdien Z, Chao BC, Gajewski E, Rao G (1991) Chemical nature of in vivo DNA base damage in hydrogen peroxide-treated mammalian cells. Arch Biochem Biophys 285: 388–390. 1654775 10.1016/0003-9861(91)90378-v

[33] Koizumi A, Weindruch R, Walford RL (1987) Influences of dietary restriction and age on liver enzyme activities and lipid peroxidation in mice. J Nutr 117: 361–367. 3031254 10.1093/jn/117.2.361

[34] Rao G, Xia E, Nadakavukaren MJ, Richardson A (1990) Effect of dietary restriction on the age-dependent changes in the expression of antioxidant enzymes in rat liver. J Nutr 120: 602–609. 2352034 10.1093/jn/120.6.602

[35] Gomi F, Matsuo M (1998) Effects of aging and food restriction on the antioxidant enzyme activity of rat livers. J Gerontol A Biol Sci Med Sci 53: B161–167. 9597038 10.1093/gerona/53a.3.b161

[36] Sohal RS, Weindruch R (1996) Oxidative stress, caloric restriction, and aging. Science 273: 59–63. 8658196 10.1126/science.273.5271.59 PMC2987625

[37] Redman LM, Ravussin E (2011) Caloric restriction in humans: impact on physiological, psychological, and behavioral outcomes. Antioxid Redox Signal 14: 275–287. 20518700 10.1089/ars.2010.3253 PMC3014770

[38] Heilbronn LK, de Jonge L, Frisard MI, DeLany JP, Larson-Meyer DE, et al (2006) Effect of 6-month calorie restriction on biomarkers of longevity, metabolic adaptation, and oxidative stress in overweight individuals: a randomized controlled trial. JAMA 295: 1539–1548. 16595757 10.1001/jama.295.13.1539 PMC2692623

[39] Yagi K (1976) A simple fluorometric assay for lipoperoxide in blood plasma. Biochem Med 15: 212–216. 962904 10.1016/0006-2944(76)90049-1

[40] Davi G, Guagnano MT, Ciabattoni G, Basili S, Falco A, et al (2002) Platelet Activation in Obese Women: Role of Inflammation and Oxidant Stress. JAMA 288: 2008–2014. 12387653 10.1001/jama.288.16.2008

[41] Tsai IJ, Croft KD, Mori TA, Falck JR, Beilin LJ, et al (2009) 20-HETE and F2-isoprostanes in the metabolic syndrome: the effect of weight reduction. Free Radical Biology and Medicine 46: 263–270. 19013235 10.1016/j.freeradbiomed.2008.10.028

[42] Roberts LJ, Morrow JD (2000) Measurement of F2-isoprostanes as an index of oxidative stress in vivo. Free Radical Biology and Medicine 28: 505. 10719231 10.1016/s0891-5849(99)00264-6

[43] Ridker PM, Rifai N, Clearfield M, Downs JR, Weis SE, et al (2001) Measurement of C-Reactive Protein for the Targeting of Statin Therapy in the Primary Prevention of Acute Coronary Events. N Engl J Med 344: 1959–1965. 11430324 10.1056/NEJM200106283442601

[44] Richelle M, Turini ME, Guidoux R, Tavazzi I, Métairon S, et al (1999) Urinary isoprostane excretion is not confounded by the lipid content of the diet. FEBS Letters 459: 259–262. 10518031 10.1016/s0014-5793(99)01259-4

[45] Gopaul N, Halliwell B, Anggård E (2000) Measurement of plasma F2-isoprostanes as an index of lipid peroxidation does not appear to be confounded by diet. Free Radic Res 33: 115–127. 10885619 10.1080/10715760000300671

[46] Codoñer-Franch P, Valls-Bellés V, Arilla-Codoñer A, Alonso-Iglesias E Oxidant mechanisms in childhood obesity: the link between inflammation and oxidative stress. Translational Research. 10.1016/j.trsl.2011.08.004 22061044

[47] Tsai WC LY, Lin CC, Chao TH, Chen JH (2004) Effects of oxidative stress on endothelial function after a high-fat meal. Clin Science 106: 315–319. 10.1042/CS20030227 14561213

[48] Bae J-H, Bassenge E, Kim K-B, Kim Y-N, Kim K-S, et al (2001) Postprandial hypertriglyceridemia impairs endothelial function by enhanced oxidant stress. Atherosclerosis 155: 517. 11254924 10.1016/s0021-9150(00)00601-8

[49] Ziccardi P, Nappo F, Giugliano G, Esposito K, Marfella R, et al (2002) Reduction of Inflammatory Cytokine Concentrations and Improvement of Endothelial Functions in Obese Women After Weight Loss Over One Year. Circulation 105: 804–809. 11854119 10.1161/hc0702.104279

[50] Wray DW, Nishiyama SK, Donato AJ, Carlier P, Bailey DM, et al. (2011) The Paradox of Oxidative Stress and Exercise With Advancing Age. Exercise and Sport Sciences Reviews 39: 68–76 10.1097/JES.1090b1013e31820d37657. 10.1097/JES.0b013e31820d7657 PMC6116907 21206280

[51] Higdon JV, Frei B (2003) Obesity and Oxidative Stress: A Direct Link to CVD? Arterioscler Thromb Vasc Biol 23: 365–367. 12639823 10.1161/01.ATV.0000063608.43095.E2

[52] Heilbronn L, Civitarese AE, Bogacka I, Smith S, Hulver M, et al (2005) Glucose tolerance and skeletal muscle gene expression in response to alternate day fasting. Obes Res 13: 574–581. 15833943 10.1038/oby.2005.61

[53] Hall KD, Jordan PN (2008) Modeling weight-loss maintenance to help prevent body weight regain. The American Journal of Clinical Nutrition 88: 1495–1503. 19064508 10.3945/ajcn.2008.26333

[54] Strychar I, Lavoie M-È, Messier L, Karelis AD, Doucet É, et al (2009) Anthropometric, Metabolic, Psychosocial, and Dietary Characteristics of Overweight/Obese Postmenopausal Women with a History of Weight Cycling: A MONET (Montreal Ottawa New Emerging Team) Study. Journal of the American Dietetic Association 109: 718–724. 19328269 10.1016/j.jada.2008.12.026

[55] Pieper C, Redman L, Racette S, Roberts S, Bhapkar M, et al (2011) Development of adherence metrics for caloric restriction interventions. Clin Trials 8: 155–164. 21385788 10.1177/1740774511398369 PMC3095229

[56] Field AE, Manson JE, Taylor CB, Willett WC, Colditz GA (2004) Association of weight change, weight control practices, and weight cycling among women in the Nurses' Health Study II. International journal of obesity and related metabolic disorders : journal of the International Association for the Study of Obesity 28: 1134–1142. 10.1038/sj.ijo.0802728 15263922

[57] Lahti-Koski M, Mannisto S, Pietinen P, Vartiainen E (2005) Prevalence of weight cycling and its relation to health indicators in Finland. Obes Res 13: 333–341. 15800292 10.1038/oby.2005.45

[58] Osborn R, Forys K, Psota T, Sbrocco T (2011) Yo-yo dieting in African American women: weight cycling and health. Ethn Dis 21: 274–280. 21942158 PMC3963267

[59] Cereda E, Malavazos AE, Caccialanza R, Rondanelli M, Fatati G, et al (2011) Weight cycling is associated with body weight excess and abdominal fat accumulation: A cross-sectional study. Clinical Nutrition 30: 718–723. 21764186 10.1016/j.clnu.2011.06.009

[60] Dulloo AG, Jacquet J, Montani J-P (2012) How dieting makes some fatter: from a perspective of human body composition autoregulation. Proceedings of the Nutrition Society 71: 379–389. 22475574 10.1017/S0029665112000225

[61] Hooper LE, Foster-Schubert KE, Weigle DS, Sorensen B, Ulrich CM, et al (2010) Frequent intentional weight loss is associated with higher ghrelin and lower glucose and androgen levels in postmenopausal women. Nutrition Research 30: 163–170. 20417876 10.1016/j.nutres.2010.02.002 PMC2992868

[62] Graci S, Izzo G, Savino S, Cattani L, Lezzi G, et al (2003) Weight cycling and cardiovascular risk factors in obesity. International journal of obesity and related metabolic disorders : journal of the International Association for the Study of Obesity 28: 65–71. 10.1038/sj.ijo.0802537 14647176

[63] Stevens VL, Jacobs EJ, Sun J, Patel AV, McCullough ML, et al (2012) Weight Cycling and Mortality in a Large Prospective US Study. American Journal of Epidemiology 175: 785–792. 22287640 10.1093/aje/kwr378

[64] Hooper LE, Foster-Schubert KE, Weigle DS, Sorensen B, Ulrich CM, et al (2010) Frequent intentional weight loss is associated with higher ghrelin and lower glucose and androgen levels in postmenopausal women. Nutr Res 30: 163–170. 20417876 10.1016/j.nutres.2010.02.002 PMC2992868

[65] Institute of Medicine (2000) Panel on Dietary Antioxidants and Related Compounds: Dietary reference intakes for vitamin C, vitamin E, selenium, and carotenoids. Washington, D.C.: National Academy Press. 506 p. p.

[66] Heilbronn L, Ravussin E (2003) Caloric restriction and aging: review of the literature and implications for studies in humans. The American Journal of Clinical Nutrition 78: 361–369. 12936916 10.1093/ajcn/78.3.361

[67] Davalos A, Gomez-Cordoves C, Bartolome B (2004) Extending applicability of the oxygen radical absorbance capacity (ORAC-fluorescein) assay. J Agric Food Chem 52: 48–54. 14709012 10.1021/jf0305231

[68] Apak R, Guclu K, Ozyurek M, Bektasoglu B, Bener M (2010) Cupric ion reducing antioxidant capacity assay for antioxidants in human serum and for hydroxyl radical scavengers. Methods Mol Biol 594: 215–239. 20072920 10.1007/978-1-60761-411-1_15

[69] Nemzer BV, Rodriguez LC, Hammond L, Disilvestro R, Hunter JM, et al (2011) Acute reduction of serum 8-iso-PGF2-alpha and advanced oxidation protein products in vivo by a polyphenol-rich beverage; a pilot clinical study with phytochemical and in vitro antioxidant characterization. Nutr J 10: 67. 21676230 10.1186/1475-2891-10-67 PMC3141640

[70] Wray DW, Nishiyama SK, Harris RA, Zhao J, McDaniel J, et al (2012) Acute reversal of endothelial dysfunction in the elderly after antioxidant consumption. Hypertension 59: 818–824. 22353612 10.1161/HYPERTENSIONAHA.111.189456 PMC3321727

