# Supplementary material for: Effect of Modest Caloric Restriction on Oxidative Stress in Women, a Randomized Trial
Source: PLoS One. 2012 Oct 5;7(10):e47079. doi: 10.1371/journal.pone.0047079 (PMC3465282; doi:10.1371/journal.pone.0047079)
Supplement: Protocol S1 — Trial Protocol. (DOCX) [file pone.0047079.s002.docx]

**Protocol S1**

**Effect of Modest Caloric Restriction on Oxidative Stress in Women, a Randomized Trial.**

It is not established to what extent caloric intake must be reduced to lower oxidative stress in humans. The aim of this study was to determine the effect of short-term, moderate caloric restriction on markers of oxidative stress in overweight and obese premenopausal women. We will conduct a randomized trial in women assign to one of two groups. One group will serve as a control group. This group will be given food containing 100% of daily caloric requirements. The second experimental group will be given a diet containing 75% of daily caloric requirements. This study is trying to determine how a low calorie diet might affect oxidative stress humans.

**Study site location and design:**

This randomized trial will be conducted at Vanderbilt University Medical Center. Eligible participants are premenopausal overweight and obese women, with inclusion criteria of: BMI more than25 kg/m^2^ and willingness to abstain from alcohol consumption for the duration of the study, complete an overnight stay in the Clinical Research Center (CRC), and come to the CRC daily to obtain study foods.

**Experimental Design:**

This study will consist of a 4 (28 days) dietary study, and 4 follow up visits (at week 2 and months 3, 6, and 12 after the dietary study).

Below is a chart of all the tests and when they will be performed.

| **Days** | **Screening 1** | **Screening 2** | **0** |
| --- | --- | --- | --- |
| Metabolic room / Cart | X |  |  |
| Weight | X | X | X |
| Height/waist/hips |  | X | X |
| Body Composition |  | X |  |
| Blood |  | X | X |
| 24Hr.Urine |  | X | X |
| Food Diary | X | X | X |
| Physical activity | X |  | X |

| **Days** | **1** | **2** | **3** | **4** | **5** | **6** | **7** | **8** | **9** | **10** | **11** | **12** | **13** | **14** |
| --- | --- | --- | --- | --- | --- | --- | --- | --- | --- | --- | --- | --- | --- | --- |
| Metabolic room / Cart |  |  |  |  |  |  |  |  |  |  |  |  |  | X |
| Weight | X | X | X | X | X | X | X | X | X | X | X | X | X | X |
| Height/waist/hips | X | X | X | X | X | X | X | X | X | X | X | X | X | X |
| Body Composition |  |  |  |  |  |  |  |  |  |  |  |  |  |  |
| Blood |  |  | X |  | X |  | X |  |  |  |  |  |  | X |
| 24Hr. Urine collection |  |  | X |  | X |  |  |  |  | X |  |  |  | X |
| Food Diary | X | X | X | X | X | X | X | X | X | X | X | X | X | X |
| Physical activity | X | X | X | X | X | X | X | X | X | X | X | X | X | X |

| Days | 15 | 16 | 17 | | 18 | 19 | 20 | | 21 | 22 | 23 | | 24 | 25 | 26 | | 27 | 28 | 29 |
| --- | --- | --- | --- | --- | --- | --- | --- | --- | --- | --- | --- | --- | --- | --- | --- | --- | --- | --- | --- |
| Metabolic room or cart |  |  |  | |  |  |  | |  |  |  | |  |  |  | |  | X |  |
| Fat Biopsy |  |  |  | |  |  |  | |  |  |  | |  |  |  | |  |  |  |
| Weight | X | X | X | | X | X | X | | X | X | X | | X | X | X | | X | X | X |
| Height/waist/hips | X | X | X | | X | X | X | | X | X | X | | X | X | X | | X |  | X |
| Body Composition | X |  |  | |  |  |  | |  |  |  | |  |  |  | |  |  | X |
| Blood |  |  |  | |  |  |  | | X |  |  | |  |  |  | |  |  | X |
| 24Hr. Urine collection |  |  | X | |  |  | X | |  | X |  | |  | X |  | | X |  | X |
| Food Diary | X | X | X | | X | X | X | | X | X | X | | X | X | X | | X | X | X |
| Physical activity | X | X | X | | X | X | X | | X | X | X | | X | X | X | | X | X | X |
| **Follow-up Period** | **2 week** | | | **3-months** | | | | **6-months** | | | | **12-months** | | | |  |  |  |  |
| Metabolic room /Cart | X | | | X | | | | X | | | | X | | | |  |  |  |  |
| Weight | X | | | X | | | | X | | | | X | | | |  |  |  |  |
| Height/waist/hips | X | | | X | | | | X | | | | X | | | |  |  |  |  |
| Body Composition | X | | | X | | | | X | | | | X | | | |  |  |  |  |
| Blood | X | | | X | | | | X | | | | X | | | |  |  |  |  |

**Weight, height, waist and hip circumference measurements:**

Body weight will be measured with a calibrated scale and height measured with a wall-mounted height meter while in street clothes with no outerwear or shoes.

**Blood Analysis:**

Pregnancy test, catecholamines, insulin, leptin, isoprostanes, carnitine, c-reactive protein, interleukin, and genetic screening. The amounts will be as follows:

| Days | -7 | 0 | 3 | 5 | 7 | 14 | 21 | 28 | 35 |
| --- | --- | --- | --- | --- | --- | --- | --- | --- | --- |
| Amount* Drawn | 42ml | 25ml | 5 ml | 5 ml | 25 ml | 30 ml | 25 ml | 30 ml | 25 ml |

| Follow-up Days | 2 weeks | 3 months | 6 months | 12 months | Total  Entire Study |
| --- | --- | --- | --- | --- | --- |
| Amount* Drawn | 32 ml | 32 ml | 32 ml | 32 ml | 315 ml (< 1 cup) |

*1 tablespoon = 15 ml; 1 teaspoon = 5 ml

**Urine Analysis:**

Collect 24-hour urines during one weekdays and one weekend day of each study period for a total of eight (10) 24-hour urine collections.

**Statistical analysis**

Daily caloric intake and resting energy expenditure are expressed as the absolute and deficit number of kilocalories per day. The baseline characteristics between treatment and control groups are compared using the non-parametric Wilcox test. The effect of energy restriction on F_2_-isoprostane was evaluated using a paired t-test on the pre- and post-diet measurements. The effects of adiposity on F_2_-isoprostane concentration at different time points were determined by a multivariate analysis of variance with repeated measures in which body fat was treated as the independent variable. Residual analysis was performed for evaluation of model adequacy and outliers. A P-value < 0.05 was used for inclusion of terms in the regression and to indicate statistical significance. Analyses were performed with STATA 11 (StataCorp, College Station, TX) and R ([www.r-project.org](http://www.r-project.org/)) and all summary statistics are presented as means ± SD.
